# Incremental economic burden associated with exudative age-related macular degeneration: a population-based study

**DOI:** 10.1186/s12913-019-4666-0

**Published:** 2019-11-12

**Authors:** Siin Kim, Sang Jun Park, Seong Jun Byun, Kyu Hyung Park, Hae Sun Suh

**Affiliations:** 1 College of Pharmacy, Pusan National University, 2, Busandaehak-ro 63beon-gil, Geumjeong-gu, Busan, Republic of Korea; 2 Department of Ophthalmology, Seoul National University College of Medicine, Seoul National University Bundang Hospital, 82, Gumi-ro 173beon-gil, Bundang-gu, Seongnam-si, Gyeonggi-do Republic of Korea; 3 School of Pharmacy, Sungkyunkwan University, 2066, Seobu-ro, Jangan-gu, Suwon-si, Gyeonggi-do Republic of Korea

**Keywords:** Age-related macular degeneration, Cost of illness, Exudative AMD, Incremental cost, Macular degeneration, Models, econometric, Ranibizumab, Propensity score

## Abstract

**Background:**

The exudative age-related macular degeneration (AMD) causes considerable healthcare costs for patients and healthcare system, which are expected to grow as the population ages. The objective of this study was to assess the incremental economic burden of exudative AMD by comparing total healthcare costs between the exudative AMD group and non-AMD group to understand economic burden related to exudative AMD.

**Methods:**

This retrospective cohort study used the National Health Insurance Service database including the entire Korean population. Exudative AMD group included individuals with at least one claim for ranibizumab and one claim using the registration code for exudative AMD (V201). Non-AMD group was defined as individuals without any claims regarding the diagnostic code of H35.3 or ranibizumab. The exudative AMD group and non-AMD group were matched using a propensity-score model. Incremental healthcare resource utilization and healthcare costs were measured during a one-year follow-up by employing econometric models: ordinary least squares (OLS) with log transformation and heteroscedastic retransformation; and generalized linear model (GLM) with a log link function and gamma distribution.

**Results:**

A total of 7119 exudative AMD patients were matched to 7119 non-AMD patients. The number of outpatient visits was higher in the exudative AMD group (*P*-value < 0.0001), while the length of hospitalization was shorter in exudative AMD group (*P*-value < 0.0001). Exudative AMD patients had total costs 2.13 times (95%CI, 2.08–2.17) greater than non-AMD group using OLS, and total costs 4.06 times (95%CI, 3.82–4.31) greater than non-AMD group using GLM. Annual incremental total costs were estimated as $5519 (OLS) and $3699 (GLM).

**Conclusions:**

Exudative AMD was associated with significantly increased healthcare costs compared to the non-AMD group. Attention is needed to manage the socioeconomic burden of exudative AMD.

## Background

Age-related macular degeneration (AMD) is a major cause of irreversible visual loss in the elderly, and can be classified as nonexudative or exudative [1]. The major risk factor for AMD is old age, and generally a progressive and irreversible visual loss occurs after the development of exudative AMD [2, 3]. Visual loss is related with substantially decreased quality of life that is comparable to that of severe systemic diseases such as cancer, stroke, and diabetes mellitus [4].

Early intervention with anti-vascular endothelial growth factor (VEGF) therapy can reduce visual loss in patients with exudative AMD, whereas it causes considerable healthcare costs for patients and healthcare system due to its high price and needs for repetitive administration accompanied by frequent outpatient visits [2, 5]. To ease the economic burden of patients with AMD, the Korean registration program for rare intractable diseases provides copayment reduction. Given that the prevalence of exudative AMD was reported to be 0.36–0.60% in 2008–2014 in Korea and the prevalence is continuously increasing as the population ages, the economic burden of exudative AMD is also expected to grow for both the individual patient and society [6–8].

However, there is a paucity of data exploring economic burden of exudative AMD. A prospective, cross-sectional study conducted in Canada, France, Germany, Spain, and the UK estimated the annual societal cost per patient to be €5300–12,445 in 2005 [9]. Among the UK cohort in this five-country cross-sectional study, the annual healthcare costs of patients with exudative AMD was more than seven times greater than that of non-AMD subjects [10]. In Korea, the annual cost of anti-VEGF therapy per patient has increased from $7947 in 2010 to $10,246 in 2014, and the total burden of anti-VEGF therapy in Korean population was expected to continue to increase by the year 2030 [7]. Those studies are limited by several factors, such as the absence of appropriate controls comparable to patients with exudative AMD in demographic and clinical characteristics, possibilities of recall bias resulting from the telephone survey, and a narrow definition of economic burden confined to the treatment costs for exudative AMD. Within the limited budget, efficient allocation of healthcare resources is crucial for sustainability of healthcare system. Burden of disease can be used as an evidence for priority in healthcare policies.

Therefore, we aimed to compare the incremental burden, which encompasses all aspects of medical expenses, in the exudative AMD group and non-AMD reference group, whose set of comorbidities is similar to the exudative AMD group, by using a nationwide claims database covering the entire Korean population.

## Methods

### Study design and data source

We conducted a retrospective cohort study using data from the National Health Insurance Service (NHIS) database from 2006 through 2013, which was the total period of available dataset. The NHIS database includes records for the entire Korean population, since a single payer program covers the entire Korean population, about 50 million persons, through either the National Health Insurance system (97%) or Medical Aid (3%) [11]. Therefore, the NHIS database contains all medical records related to medical claims made in Korea. We analyzed 2 different NHIS datasets simultaneously in the present study: the NHIS–customized database, which consists of all patients with AMD extracted from the whole NHIS database covering the entire Korean population, and the NHIS–National Sample Cohort (NSC) database, which consists of a validated and representative sample of 1,025,340 Korean residents (approximately 2.2% of Korean population) [12]. As the NHIS did not allow us to access the whole database to set the non-AMD population, we used the NHIS-NSC database instead. Both datasets were built by NHIS, and only authorized researchers were able to visit the analysis center and access an onsite server for the purpose of analysis.

### Study population

The provided NHIS-customized database consisted of all patients with degeneration of macula having diagnostic code of H35.3 among the entire Korean population. Of these, we defined patients with incident exudative AMD when individuals with at least one claim for ranibizumab and one claim using the registration code for exudative AMD (V201) between August 1, 2010 and December 31, 2012. Korean registration program for rare intractable diseases was initiated in 2006 and covers 138 rare intractable diseases including exudative AMD for copayment reduction (up to 10%). To be registered for copayment reduction, ophthalmologist should confirm the diagnosis of exudative AMD according to the NHIS diagnostic criteria as follows: (1) a dilated fundus examination using indirect ophthalmoscopy, (2) optical coherence tomography, and (3) fluorescein angiography to confirm the exudative AMD-related macular pathologic features. The NHIS reviews the eligibility at the first registration and verifies the accuracy and reliability of the exudative AMD diagnosis based on the test results described above and doctor’s note. After registration, all exudative AMD-related claims contain the registration code V201 in addition to the diagnostic code for exudative AMD. Detailed information was reported elsewhere [6]. As the NHIS scheme covers only single anti-VEGF agent during the study period, ranibizumab since August 2009 (aflibercept has been covered since November 2014), we included only ranibizumab in the analysis. The first date of the claim for ranibizumab was designated as the index date. The index date had to precede the first time a claim was made with registration code V201 or be with 90 days of the first claim with registration code V201. In order to identify patients with incident exudative AMD, individuals were excluded if they had any claims with ranibizumab or a registration code V201 between January 1, 2006 and July 31, 2010.

We set the non-AMD group using the NHIS-NSC database. The non-AMD group was defined as individuals without any claims regarding the diagnostic code of H35.3 (‘degeneration of macula and posterior pole’), which includes H35.30 (‘nonexudative AMD’) and H35.31 (‘exudative AMD’) or regarding ranibizumab during the whole study period.

Of these individuals in the both groups, we excluded individuals having a history or new diagnosis of malignant neoplasms (diagnostic codes of C00-C97) and stroke (hospitalization with diagnostic codes of I60–I64) during the entire study period, because these diseases generally involve enormous costs that can affect the healthcare costs of exudative AMD [13].

### Matching non-AMD group to the exudative AMD group based on propensity scores

We preliminarily selected 10 to 20 matching-candidates as the non-AMD group to each exudative AMD patient according to their age group. Each of the matching-candidates had to visit a healthcare facility on the same date with the index date of matched-exudative AMD patient. We assigned the same index dates to these matching-candidates as their matched-exudative AMD patient. Then, we computed the propensity scores in the exudative AMD group and their matching-candidates using a logistic regression model and included the following covariates: the index date; age group, sex, type of insurance, income level, residence and disability at the index date; Charlson Comorbidity Index (CCI) score, comorbidities (i.e. myocardial infarction, congestive heart failure, and dementia), length of hospitalization, number of outpatient visits and number of emergency department visits during the 1 year before the index date. Square and interaction terms were also included. To calculate CCI score, all diagnostic codes during the 1 year before the index date were analyzed according to the Quan’s coding algorithms [14]. Thereafter, we selected 1 non-AMD individual among matching-candidates to each exudative AMD patient using estimated propensity scores. The propensity score model was regarded as being correctly specified if the baseline covariates were well-balanced between the exudative AMD and non-AMD groups in terms of standardized differences (< 10%) and *P*-values (≥0.05), which measure the differences between summary statistics of baseline covariates among two groups [15, 16]. The discriminatory power of the propensity score model was measured as a *c*-statistic.

### Outcome measures

The exudative AMD and non-AMD groups were followed for 1 year to measure healthcare resource utilization and healthcare costs. Annual healthcare resource utilization per patient was assessed including the total length of hospitalization, number of hospitalizations, number of outpatient visits, and number of emergency department visits. From the perspective of Korean payer, healthcare costs incorporated direct medical costs incurred by both the insurer and the patient. Using a gross-costing approach, annual healthcare cost per patient was calculated as the sum of all expenses incurred during the period from 3 months before the index date to 1 year after the index date. This analysis began 3 months prior to the index date so as to include pre-diagnosis costs, which occur with regard to a diagnosis of exudative AMD (e.g. retina fundus photo and fundus fluorescein angiography, which are essential in the diagnosis of exudative AMD). Healthcare costs included all expenses identifiable in healthcare claims, such as outpatient visit, hospitalization, medication, medical devices, and laboratory test. We categorized healthcare costs into pre-diagnosis costs, AMD-related costs, ophthalmology costs, and non-ophthalmology costs. The total burden of exudative AMD for the Korean population was calculated according to the following formula:

$$ \mathrm{Total}\ \mathrm{burden}\ \mathrm{of}\ \mathrm{exudative}\ \mathrm{AMD}=\left(\mathrm{annual}\ \mathrm{healthcare}\ \mathrm{cost}\ \mathrm{per}\ \mathrm{patient}\right)\times \left(\mathrm{projected}\ \mathrm{number}\ \mathrm{of}\ \mathrm{Korean}\ \mathrm{patients}\ \mathrm{with}\ \mathrm{exudative}\ \mathrm{AMD}\ \mathrm{in}\ 2014\right) $$

We projected the number of Korean patients with exudative AMD in 2014 using the direct standardization method based on the 2008–2012 Korean data estimated in the epidemiologic study for exudative AMD in Korea and the information of population structure provided by the Korean Statistical Information Service [6, 17]. Healthcare costs were measured in Korean Won (KRW) and converted to United States Dollars (USD) using the average 2017 exchange rate (1 USD = 1130.84 KRW).

### Statistical analysis

We compared baseline characteristics, including demographic and clinical characteristics, annual healthcare resource utilization, and annual healthcare costs between the exudative AMD and non-AMD groups. Continuous variables were tested using the paired t-test, while categorical variables were tested with McNemar’s test.

As most cost data has a positively skewed distribution, it is difficult to obtain unbiased and precise estimates of incremental costs by calculating mean differences or running a simple ordinary least squares (OLS) [18]. Historically, an OLS for log-scale or a generalized linear model (GLM) with log links analysis can be used to address observed skewed distribution in the analysis. To choose the type of model, we examined the kurtosis and heteroscedasticity of the residuals from the log-scale analysis, according to a previously developed algorithm [18]. Given that the log-scale residuals were both leptokurtic and heteroscedastic, following two models were compared to assess the incremental cost of exudative AMD group compared with non-AMD group: (1) OLS with a log transformation and heteroscedastic retransformation and (2) GLM with a log link function and gamma distribution. We adjusted for age group (5-year interval), sex, type of insurance, residence, disability, CCI score, and baseline healthcare resource utilization in both models. For the heteroscedastic retransformation, a variance function was estimated for the independent variables suspected of causing heteroscedasticity, such as age group, type of insurance, CCI score, and baseline healthcare resource utilization [19].

SAS version 9.4 (SAS Institute Inc., Cary, NC, USA) and STATA SE 14.0 was used for statistical analysis. Institutional Review Board (IRB)/Ethics Committee approval was obtained by the Seoul National University Bundang Hospital (X-1710-429-907) and the study complied with the guidelines of the Declaration of Helsinki.

## Results

**Fig. 1:**
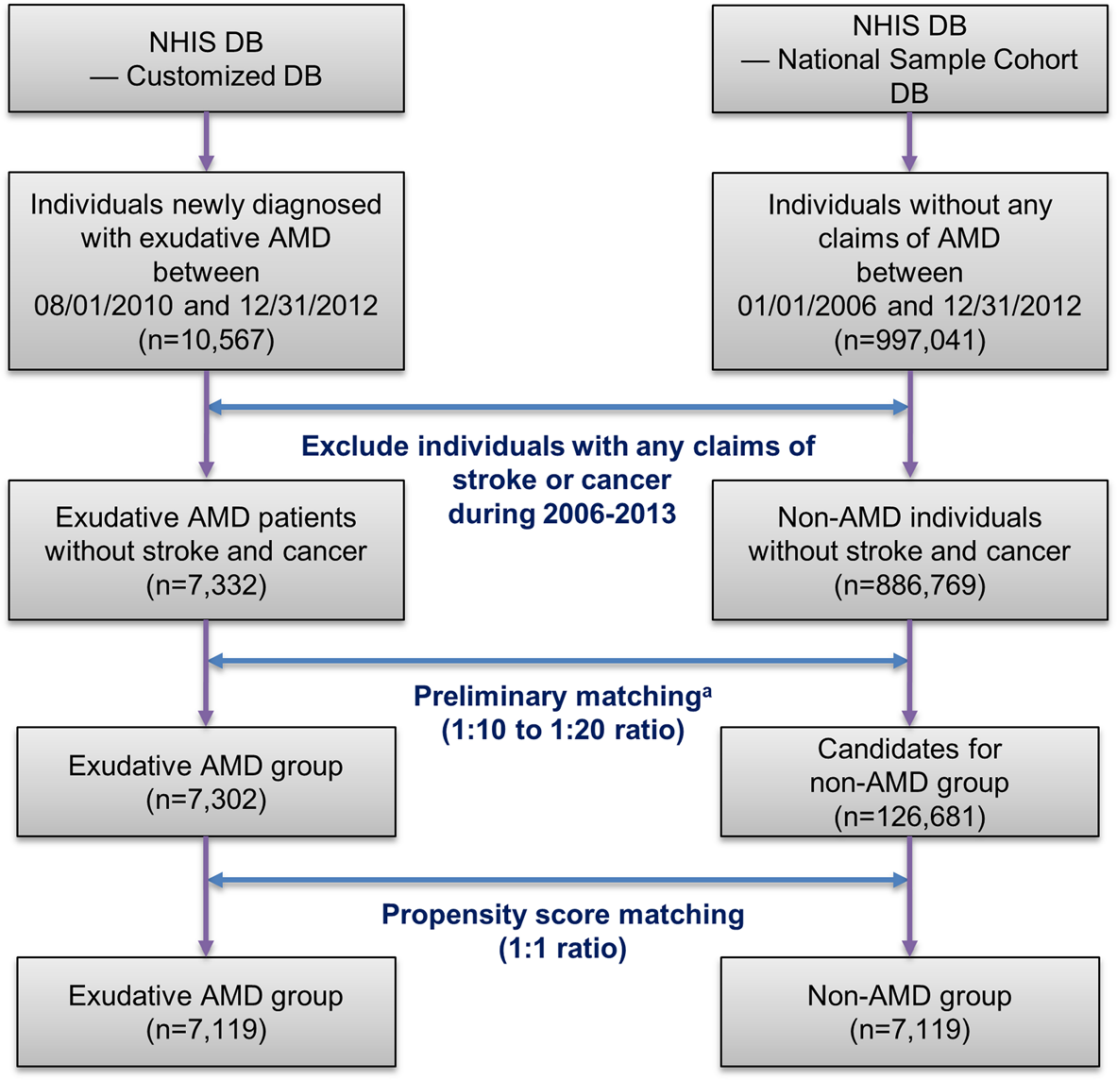
Sample selection flow. AMD, age-related macular degeneration; DB, database; NHIS, National Health Insurance Service. ^a^Candidates for the non-AMD group were preliminarily matched to the exudative AMD group in a 1:10 to 1:20 ratio by age group and date of healthcare facility visits

A total of 7119 individuals were identified as incident exudative AMD patients who met the eligibility criteria; these were matched to 7119 individuals who constituted the non-AMD group (Fig. 1). The exudative AMD and non-AMD matching-candidates differed significantly in most of their baseline characteristics (Additional file 1: Table S1). However, after propensity score-based matching, baseline characteristics between the exudative AMD and non-AMD groups were well-balanced in terms of standardized differences and *P*-values (Table 1). The *c*-statistic of the propensity score model was 0.663.

**Table 1:** Baseline charateristics of study population after propensity score matching

Characteristic	Exudative AMD group (*n* = 7119)	Non-AMD group (*n* = 7119)	*P*-value	Standardized difference (%)
Male, No. (%)	4099 (57.58)	4134 (58.07)	0.41	−0.99
Age, No. (%), years				
< 45	10 (0.14)	7 (0.10)	0.59	1.16
45–49	65 (0.91)	65 (0.91)		0.00
50–54	438 (6.15)	442 (6.21)		−0.25
55–59	761 (10.69)	746 (10.48)		0.68
60–64	1092 (15.34)	1122 (15.76)		−1.16
65–69	1334 (18.74)	1377 (19.34)		−1.53
70–74	1428 (20.06)	1376 (19.33)		1.84
75–79	1110 (15.59)	1104 (15.51)		0.22
80–84	607 (8.53)	620 (8.71)		−0.64
≥ 85	274 (3.85)	260 (3.65)		1.05
Income level, No. (%)^a^				
0	277 (3.89)	256 (3.60)	0.66	1.53
1	562 (7.89)	555 (7.80)		0.33
2	445 (6.25)	433 (6.08)		1.25
3	421 (5.91)	419 (5.89)		0.71
4	420 (5.90)	456 (6.41)		0.08
5	466 (6.55)	493 (6.93)		−2.12
6	545 (7.66)	547 (7.68)		−1.52
7	640 (8.99)	624 (8.77)		−0.08
8	750 (10.54)	744 (10.45)		0.77
9	1074 (15.09)	1109 (15.58)		0.29
10	1519 (21.34)	1483 (20.83)		−1.36
Insurance, No. (%)				
NHI program	6842 (96.11)	6863 (96.40)	0.54	−1.53
Medical aid program	277 (3.89)	256 (3.60)		1.53
Residence, No. (%)				
Seoul (Capital city)	1649 (23.16)	1611 (22.63)	0.79	1.26
Metropolitan city	1738 (24.41)	1720 (24.16)		0.58
Others	3732 (52.42)	3788 (53.21)		−1.58
Charlson comorbidity index, mean (SD)	0.59 (0.58)	0.59 (0.56)	0.96	0.00
Length of hospitalization during the baseline period, mean (SD), days	2.41 (14.62)	2.64 (13.94)	0.22	−1.47
Number of outpatient visits during the baseline period, mean (SD)	30.96 (28.22)	30.97 (28.39)	0.98	−0.02
Number of emergency department visits during the baseline period, mean (SD)	0.09 (0.46)	0.10 (0.40)	0.76	−0.32

*AMD* age-related macular degeneration, *NHI* National Health Insurance, *SD* standard deviation

^a^Income was categorized according to the insurance contribution. Level 10 denotes the highest insurance contribution (i.e. the highest income) and level 1 denotes the lowest insurance contribution (i.e. the lowest income) within NHI program. Level 0 denotes individuals covered by Medical aid program

**Fig. 2:**
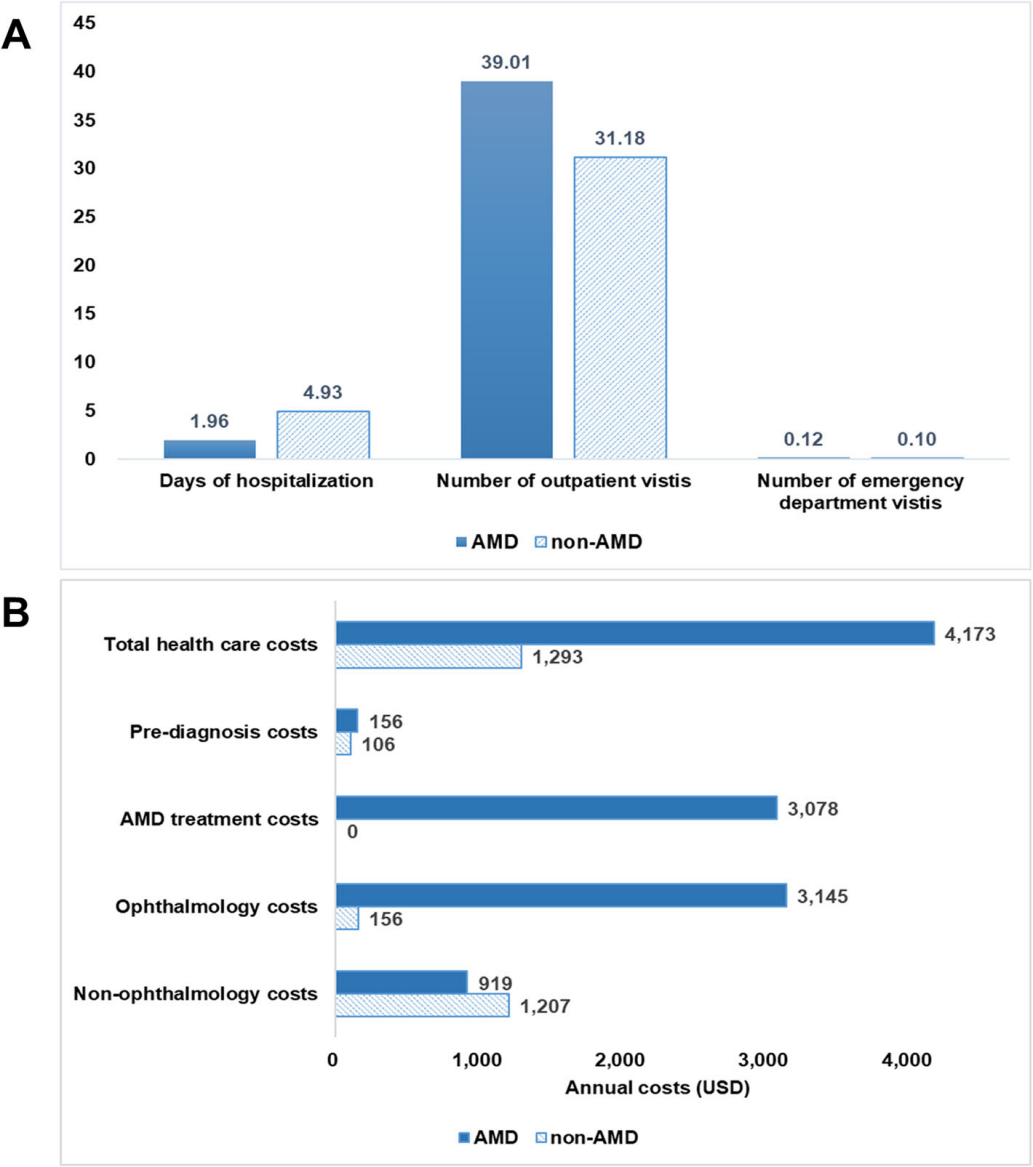
**a** Descriptive analysis of annual healthcare resource utilization during a 1-year follow-up period. **b** Descriptive analysis of annual healthcare costs during a 1-year follow-up period. AMD, age-related macular degeneration; USD, United States Dollar

Figure 2a shows that the exudative AMD group had a greater number of outpatient visits relative to the non-AMD group (mean 39.01 vs. 31.18; *P*-value < 0.0001), while the total length of hospitalization was shorter in the exudative AMD group (mean 1.96 days vs. 4.93 days; *P*-value < 0.0001) during the follow-up period. Ophthalmology costs, especially AMD treatment costs, greatly contributed to the total healthcare costs in the exudative AMD group (Fig. 2b). As noted earlier, cost data were highly skewed in both groups.

Results of the OLS analysis showed that the total healthcare cost was 2.13 times higher in the exudative AMD group relative to the non-AMD group (95% confidence interval [CI]; 2.08–2.17) (Table 2).

**Table 2:** Results of econometric models of healthcare costs associated with exudative AMD

Variables	Ordinary least square		Generalized linear model	
	Beta (95% CI)	*P*-value	Beta (95% CI)	*P*-value
AMD	2.13 (2.08 to 2.17)	< 0.001	1.40 (1.34 to 1.46)	< 0.001
Age group	−0.02 (− 0.03 to − 0.00)	0.016	0.06 (0.04 to 0.07)	< 0.001
Sex	0.13 (0.09 to 0.18)	< 0.001	0.05 (−0.01 to 0.11)	0.102
Insurance	0.01 (−0.04 to 0.05)	0.769	0.05 (−0.01 to 0.10)	0.097
Residence	−0.00 (− 0.03 to 0.02)	0.908	0.00 (− 0.03 to 0.04)	0.805
Disability	0.13 (0.06 to 0.20)	< 0.001	0.25 (0.17 to 0.34)	< 0.001
Charlson comorbidity index	0.22 (0.18 to 0.27)	< 0.001	0.21 (0.15 to 0.26)	< 0.001
Length of hospitalization	0.01 (0.01 to 0.01)	< 0.001	0.01 (0.01 to 0.01)	< 0.001
Number of outpatient visits	0.01 (0.01 to 0.02)	< 0.001	0.01 (0.01 to 0.01)	< 0.001
Number of ED visits	0.02 (−0.03 to 0.08)	0.426	0.05 (− 0.02 to 0.12)	0.179

*AMD* age-related macular degeneration, *CI* confidence interval, *ED* emergency department

The GLM analysis showed that the total healthcare cost was 4.06 times (the exponential of 1.40) higher in the exudative AMD group relative to the non-AMD group (95% CI 3.82–4.31). After retransformation, the annual incremental total cost per patient with exudative AMD was estimated as $5519 using the OLS model and $3699 using the GLM model, which were greater than the observed differences (Table 3).

**Table 3:** Annual incremental healthcare costs associated with exudative AMD in USD

Costs	Observed differences	Ordinary least square	Generalized linear model
Total costs	+ 2880	+ 5519	+ 3699
Pre-diagnosis costs	+ 50	+ 79	+ 56
AMD treatment costs	+ 3078	N/A^a^	N/A^a^
Ophthalmology costs	+ 2988	+ 7419	+ 3010
Non-ophthalmology costs	−288	+ 42	−39

AMD, age-related macular degeneration; N/A, not applicable; USD, United States Dollar

^a^Not applicable because AMD treatment costs are totally zero in non-AMD group

The estimated total burden of exudative AMD in the Korean population was $368,063,007 in 2014 ($3699 [the annual healthcare cost per patient, estimated via GLM]) * (99,503 [projected number of Korean adults aged 40 or over having exudative AMD]).

## Discussion

This study found that patients with exudative AMD had significantly higher medical costs in comparison to carefully-matched non-AMD reference group in the era of anti-VEGF treatment. Understandably, treatment costs related to exudative AMD contributes the majority of the total healthcare costs in the exudative AMD group and, however, incremental ophthalmology-related cost observed in the exudative AMD group was partly compensated by incremental non-ophthalmology-related cost in the non-AMD group.

Two studies reported the cost of exudative AMD before the era of anti-VEGF treatment. Cruess et al. reported that the annual incremental cost of a patient with bilateral exudative AMD relative to a control subject ranged from €2965 to €7887 (equivalent to $3347 to $8902 in reference to the average 2017 exchange rate) in 2005 using the prospective data collected in five countries [9]. Similar to our study, they presented that patients suffering exudative AMD had higher vision-related costs and lower non-vision-related costs than controls. In addition, Bonastre et al. also estimated that the annual costs of AMD management in four European countries ranged from €10,816 to €13,073 in 2001 [20]. In this study, cost of photodynamic therapy accounted for 84–94% of total cost of AMD management. Despite treatment strategies in these studies did not hold anymore in the era of anti-VEGF treatment, they endorsed the heavy burden of exudative AMD even before the paradigm shift of exudative AMD treatment.

In Korea, Jeong et al. previously estimated the annual costs per patient associated with exudative AMD to be $10,246 in 2014, which is 2–3 times higher than our estimates [7]. The difference may be largely due to the change in reimbursement criteria on the maximum number of ranibizumab injections allowable per year. In January 2013, the annual maximum number of injections was expanded from 5 injections per eye to 10 injections per patient. Thereafter, 14 injections per patient started being covered in November 2014. Considering our analysis was based on the data between 2010 and 2013 whereas Jeong et al. used the data in 2014, the estimated number of ranibizumab injections per patient might be larger in Jeong et al. compared with our study. In fact, Jeong et al. reported that 77% of patients received ranibizumab more than 4 times a year and 35% of patients received ranibizumab more than 10 times a year. In other retrospective studies which used Korean hospital data between September 2009 and January 2014, a similar analysis period with our study, the average number of ranibizumab injections were reported as 4.0–4.5 per year [21, 22].

Considering that the general population also pays healthcare costs according to their own health needs, measuring costs without appropriate controls may exaggerate the true burden of the disease. By defining the control as the non-healthy population, with a similar severity of comorbidities to the patients with exudative AMD, we adopted a conservative approach in measuring the burden of exudative AMD. Moreover, we did not include uninsured treatments, which may have led to an underestimation of the burden of exudative AMD. In fact, optical coherence tomography (OCT) is one of the most important tests performed to assess pharmacological response and disease progress in patients with exudative AMD, but it was not insured until 2015 in Korea and was not considered in our analysis. Generally, OCT is performed at every AMD-related visit in Korea, costing about $50–$150 for each service.

In most circumstances, cost data has some or all of the following properties: (1) nonnegative outcomes; (2) a nontrivial fraction of zero outcomes; and (3) positively skewed outcomes [18]. If there is a substantial fraction of zero outcomes, a two-part model or sample selection model can be used to address the zero mass problem [23]. In this study, there was no significant impact from zero mass problems because only 0.58% of study population had zero costs for total healthcare costs. Therefore, we did not use a two-part model or sample selection model, although we did add 1 KRW to all cost data to enable log transformations. According to the previously developed algorithm [18], we compared the results from the OLS analysis with log transformation followed by heteroscedastic retransformation and GLM analysis with a log link function and gamma distribution in terms of their performance. We found that there were some difficulties in the estimation of a variance function for heteroscedastic retransformation in the OLS analysis. Although we identified independent variables suspected of causing heteroscedasticity, there was a possibility that some unmeasured confounders existed and resulted in a residual bias in the estimation of the variance function. Contrary to the OLS analysis, the GLM analysis had no retransformation issue because it could directly provide the expected mean by exponentiation without estimating the variance function. For this reason, we concluded that GLM analysis might provide more precise estimates than OLS.

To our knowledge, this is the first study to explore the incremental economic burden of exudative AMD group relative to a non-AMD reference group using a nationwide database. We tried to minimize the effect of confounders by comparing the exudative AMD and non-AMD groups, which had similar distributions of baseline covariates after being matched by propensity score. Moreover, we used a previously developed algorithm to enable more elaborate methodology for cost analysis, addressing the widespread problem of skewed distributions in medical expenditure studies.

A high economic burden of disease could be a threat to sustainability of healthcare system. Therefore, understanding the costs of disease is necessary to justify and prioritize healthcare policies, and can help decision makers find out an efficient way of allocating the limited resources. In this respect, this study may provide further evidence for the costs and resource utilization related with exudative AMD, which could be used for assessing impact of healthcare policies and cost-effectiveness of certain interventions.

Several limitations of this study should be considered. Since we used claims data, uninsured examinations (e.g. OCT and fundus indocyanine green angiography), the use of treatments for an unapproved indication, or over-the-counter agents were not included in the cost analysis. These facts might have led to an underestimation of exudative AMD treatment costs and the incremental total costs of exudative AMD. Further, exudative AMD was defined based on diagnosis and ranibizumab codes, which may not have been specific enough to prevent any misclassifications in the study population. However, given that most patients are treated with ranibizumab once they are diagnosed with exudative AMD, this misclassification issue is likely to be negligible. Since ranibizumab began to be covered in 2009, the reimbursement criteria have changed to increase the maximum number of ranibizumab injections allowable per year. While this change may affect the utilization pattern of ranibizumab, we were unable to address this detail in this study. Further studies are warranted to explore the economic burden of exudative AMD in relation to change in reimbursement criteria by using more recent data. Finally, exudative AMD can cause an economic burden beyond medical costs. Therefore, non-medical costs, such as productivity loss, should be explored in future studies.

## Conclusions

Exudative AMD was associated with significantly increased healthcare costs relative to those of a non-AMD group with similar demographic and clinical characteristics. A GLM analysis showed that patients with exudative AMD are estimated to cost an additional $3699 annually. Since the prevalence of exudative AMD is expected to grow continuously, policy actions are needed to manage the socioeconomic burden of exudative AMD.

## Supplementary information

**Additional file 1: Table S1.** Baseline characteristics of study population before propensity score matching. Summary of baseline characteristics of exudative AMD group and non-AMD matching-candidates before propensity score matching.

## Data Availability

The data that support the findings of this study are available from the National Health Insurance Service of Korea (https://nhiss.nhis.or.kr/bd/ab/bdaba000eng.do) but restrictions apply to the availability of these data, which were used under license for the current study, and so are not publicly available. Data are however available from the authors upon reasonable request and with permission of the National Health Insurance Service of Korea.

## Abbreviations

AMD: Age-related macular degeneration
CCI: Charlson Comorbidity Index
CI: Confidence interval
ED: Emergency department
GLM: Generalized linear model
IRB: Institutional Review Board
KRW: Korean Won
NHIS: National Health Insurance Service
NSC: National Sample Cohort
OCT: Optical coherence tomography
OLS: Ordinary least squares
SD: Standard deviation
USD: United States Dollars
VEGF: Vascular endothelial growth factor
