# Supplementary material for: Incremental economic burden associated with exudative age-related macular degeneration: a population-based study
Source: BMC Health Serv Res. 2019 Nov 12;19:828. doi: 10.1186/s12913-019-4666-0 (PMC6852978; doi:10.1186/s12913-019-4666-0)
Supplement: Supplementary file 1 — Additional file 1: Table S1. Baseline characteristics of study population before propensity score matching. Summary of baseline characteristics of exudative AMD group and non-AMD matching-candidates before propensity score matching. [file 12913_2019_4666_MOESM1_ESM.docx]

Table S1 Baseline characteristics of study population before propensity score matching

| **Characteristics** | **Exudative AMD group (n=7,299)** | **Non-AMD group (n=126,657)** | ***P*-value** | **Standardized difference (%)** |
| --- | --- | --- | --- | --- |
| Male, No. (%) | 4,197 (57.50) | 49,883 (39.38) | <0.0001 | 36.87 |
| Age, No. (%), years |  |  |  |  |
| <45 | 10 (0.14) | 200 (0.16) | <0.0001 | -0.52 |
| 45-49 | 67 (0.92) | 1,340 (1.06) |  | -1.41 |
| 50-54 | 445 (6.10) | 8,900 (7.03) |  | -3.76 |
| 55-59 | 776 (10.63) | 15,519 (12.25) |  | -5.09 |
| 60-64 | 1,113 (15.25) | 22,256 (17.57) |  | -6.27 |
| 65-69 | 1,360 (18.63) | 26,509 (20.93) |  | -5.78 |
| 70-74 | 1,470 (20.14) | 22,942 (18.11) |  | 5.16 |
| 75-79 | 1,149 (15.74) | 15,130 (11.95) |  | 10.99 |
| 80-84 | 626 (8.58) | 8,454 (6.67) |  | 7.20 |
| ≥85 | 283 (3.88) | 5,407 (4.27) |  | -1.97 |
| Income level, No. (%)^a^ |  |  |  |  |
| 0 | 457 (6.26) | 9,248 (7.30) | <0.0001 | -4.14 |
| 1 | 562 (7.70) | 10,655 (8.41) |  | -2.61 |
| 2 | 445 (6.10) | 8,195 (6.47) |  | -1.52 |
| 3 | 421 (5.77) | 7,584 (5.99) |  | -0.94 |
| 4 | 420 (5.75) | 7,750 (6.12) |  | -1.57 |
| 5 | 466 (6.38) | 8,683 (6.86) |  | -1.93 |
| 6 | 545 (7.47) | 9,720 (7.67) |  | -0.76 |
| 7 | 640 (8.77) | 10,970 (8.66) |  | 0.39 |
| 8 | 750 (10.28) | 13,479 (10.64) |  | -1.18 |
| 9 | 1,074 (14.71) | 17,813 (14.06) |  | 1.85 |
| 10 | 1,519 (20.81) | 22,560 (17.81) |  | 10.05 |
| Insurance, No. (%) |  |  |  |  |
| NHI program | 7,021 (96.19) | 117,409 (92.70) | <0.0001 | 15.42 |
| Medical aid program | 277 (3.80) | 9,248 (7.30) |  | -15.33 |
| Missing value | 1 (0.01) |  |  |  |
| Residence, No. (%) |  |  |  |  |
| Seoul (Capital city) | 1,687 (23.11) | 23,897 (18.87) | <0.0001 | 10.43 |
| Metropolitan city | 1,782 (24.41) | 31,275 (24.69) |  | -0.65 |
| Others | 3,830 (52.47) | 71,485 (56.44) |  | -7.98 |
| Charlson comorbidity index, mean (SD) | 0.59 (0.58) | 0.61 (0.61) | <0.0001 | -0.26 |
| Length of hospitalization during the baseline period, mean (SD), days | 2.44 (14.8) | 6.19 (34.66) | <0.0001 | -18.53 |
| Number of outpatient visits during the baseline period, mean (SD) | 30.96 (28.22) | 27.14 (27.95) | <0.0001 | 8.42 |
| Number of emergency department visits during the baseline period, mean (SD) | 0.09 (0.46) | 0.11 (0.93) | 0.18 | -0.63 |
| AMD, age-related macular degeneration; NHI, National Health Insurance; SD, standard deviation.  ^a^Income was categorized according to the insurance contribution. Level 10 denotes the highest insurance contribution (i.e. the highest income) and level 1 denotes the lowest insurance contribution (i.e. the lowest income) within NHI program. Level 0 denotes individuals covered by Medical aid program. | | | | |
